# Advances in DNA, histone, and RNA methylation mechanisms in the pathophysiology of alcohol use disorder

**DOI:** 10.3389/adar.2023.10871

**Published:** 2023-02-15

**Authors:** Tara M. Cruise, Kumar Kotlo, Emir Malovic, Subhash C. Pandey

**Affiliations:** ^1^ Center for Alcohol Research in Epigenetics, Department of Psychiatry, University of Illinois at Chicago, Chicago, IL, United States; ^2^ Jesse Brown Veterans Affairs Medical Center, Chicago, IL, United States

**Keywords:** alcohol use disorder, brain, epigenetics, DNA methylation, histone methylation, RNA methylation

## Abstract

Alcohol use disorder (AUD) has a complex, multifactorial etiology involving dysregulation across several brain regions and peripheral organs. Acute and chronic alcohol consumption cause epigenetic modifications in these systems, which underlie changes in gene expression and subsequently, the emergence of pathophysiological phenotypes associated with AUD. One such epigenetic mechanism is methylation, which can occur on DNA, histones, and RNA. Methylation relies on one carbon metabolism to generate methyl groups, which can then be transferred to acceptor substrates. While DNA methylation of particular genes generally represses transcription, methylation of histones and RNA can have bidirectional effects on gene expression. This review summarizes one carbon metabolism and the mechanisms behind methylation of DNA, histones, and RNA. We discuss the field’s findings regarding alcohol’s global and gene-specific effects on methylation in the brain and liver and the resulting phenotypes characteristic of AUD.

## Introduction

The development of alcohol use disorder (AUD) involves a self-perpetuating cycle of drinking and withdrawal. When an individual consumes alcohol, their reward pathways are stimulated, and they experience reductions in anxiety (1, 2). Chronic alcohol users suffer from cravings and elevated anxiety during withdrawal but can attenuate these symptoms by further drinking (2,3). Many of these behavioral patterns of alcohol consumption and withdrawal, as well as others implicated in the phenotypes of intoxication and addiction, occur as the result of epigenetic changes induced by alcohol (3,4). Particularly, methylation of DNA, histones, and possibly RNA have implications in the underlying pathophysiology of AUD. In this review, we provide an overview of one-carbon metabolism and the process of methylation itself. We discuss and integrate the field’s findings regarding alcohol’s global and gene-specific effects on DNA and histone methylation in both the liver and the brain and how these changes underlie the pathophysiology of AUD (4). Finally, we provide a discussion of RNA methylation, which remains understudied in alcohol addiction. By collectively examining the field’s current knowledge of RNA methylation in conjunction with DNA and histone methylation, we hope this review will provide several potential research avenues on how alcohol consumption affects genomic methylation mechanisms that underlie the neurobiology of AUD.

## One-carbon metabolism and regulation of target methylation

**FIGURE 1:**
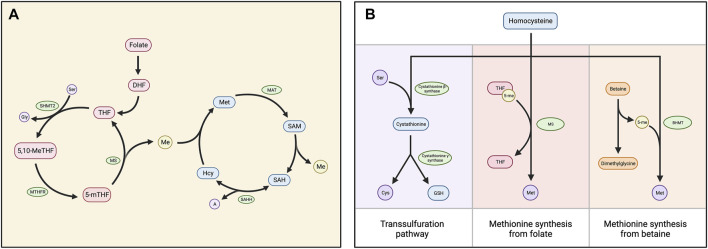
**(A)** Diagram depicting the folate and methionine cycles, which comprise the central mechanism of one carbon metabolism. Dietary folate is reduced to dihydrofolate (DHF) and then to tetrahydrofolate (THF), which is converted into 5,10-methylene THF (5,10-MeTHF) by serine hydroxymethyltransferase (SHMT2). 5,10-meTHF is converted to 5-methyl THF (5-mTHF) by methylenetetrahydrofolate reductase (MTHFR). Methionine synthase (MS) then transfers the methyl group from 5-mTHF to homocysteine (Hcy), generating THF and methionine (Met). Methionine adenosyltransferase (MAT) converts methionine into S-adenosylmethionine (SAM), which functions as a carbon donor in methylation reactions. This produces S-adenosylhomocysteine (SAH), which is cleaved into homocysteine (Hcy) and adenine (A) by SAH hydrolase (SAHH). **(B)** Chart displaying three alternatives for the processing of homocysteine. The primary mechanism is the folate cycle, in which homocysteine is converted into methionine *via* the MS-catalyzed transfer of a methyl group from 5-meTHF. Homocysteine can also be converted into methionine using betaine: Betaine-homocysteine methyltransferase (BHMT) catalyzes the transfer of a methyl group from betaine to homocysteine, generating methionine and dimethylglycine as a biproduct. Alternatively, the transsulfuration pathway processes homocysteine without converting it into methionine. Cystathionine β-synthase condenses homocysteine and serine (Ser) into cystathionine, which is cleaved into free cysteine and glutathione (GSH) by cystathionine β-synthase.

Methylation depends on the availability of S-adenosylmethionine (SAM), the universal methyl group donor. SAM is generated by the methionine cycle, a process central to one-carbon metabolism (5). In this cycle, methionine adenosyltransferase (MAT) adenylates methionine using ATP, producing SAM (5–7). SAM contains a methyl group that can be transferred to an acceptor substrate in methylation reactions, leaving S-adenosylhomocysteine (SAH) (5). SAH hydrolase (AHCY) then reversibly converts SAH into adenine and homocysteine (5, 7). However, because the reverse reaction is thermodynamically favored, adenine and homocysteine must be efficiently removed for the reaction kinetics to favor the forward reaction (5–9). The primary process for the removal of these products is the folate cycle, in which dietary folate is used to convert homocysteine into methionine, the precursor of SAM (5–7) (Figure 1A). Betaine can also be used to generate methionine from homocysteine in a process limited to the liver and kidneys (5, 6). Alternatively, the transsulfuration pathway, which is also very active in the liver, removes homocysteine without regenerating methionine (5, 9) (Figure 1B).

In addition to the reaction kinetics, feedback mechanisms regulate the efficiency of one-carbon metabolism, determining the availability of SAM and the subsequent potential for methylation reactions, called the “methylation index.” Some of these feedback mechanisms increase the methylation index. For example, glycine N-methyltransferase (GNMT), an enzyme that reduces the SAM/SAH ratio, is inhibited by 5-methyltetrahydrofolate (5-mTHF) (6, 7). Other feedbacks decrease the methylation index. For example, SAM allosterically inhibits methylenetetrahydrofolate reductase (MTHFR), while serine hydroxymethyltransferase 2 (SHMT2) sequesters 5-mTHF (6, 10). Additionally, SAH serves as a competitive inhibitor of transmethylation reactions by binding to the catalytic region of SAM-dependent methyltransferases with high affinity (5, 8, 10).

## DNA methylation and demethylation pathways

**FIGURE 2:**
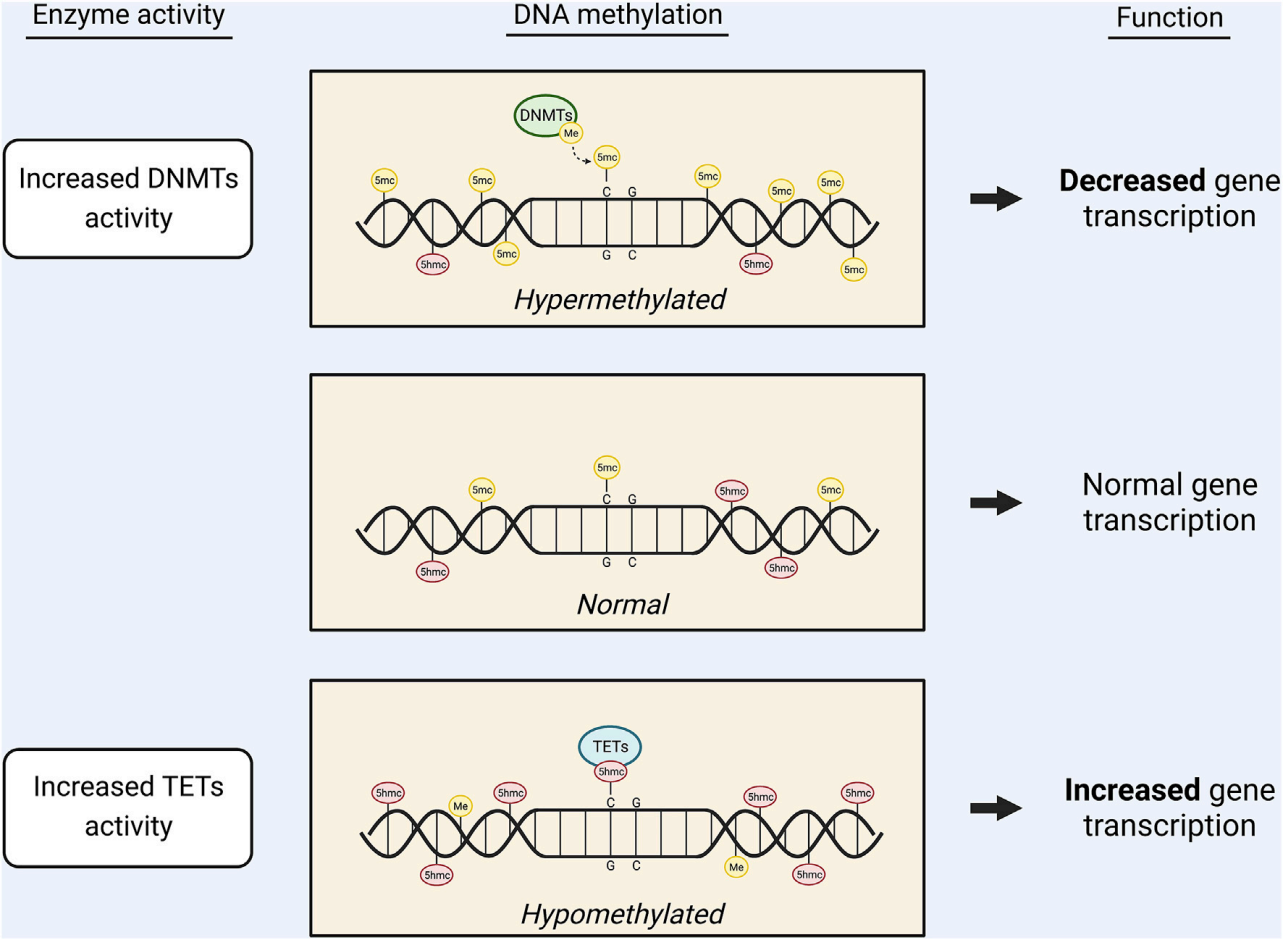
Graphic displaying the effects of DNA methylation on gene transcription. The availability of 5mc or 5hmc marks on the DNA regulates gene transcription. Increased activity of DNMTs leads to an increase in 5mc marks and, subsequently, decreased gene expression. Conversely, increased activity of TETs leads to conversion of 5mc marks into 5hmc marks, causing increased gene expression.

DNA methylation is catalyzed by DNA methyltransferases (DNMTs), which transfer methyl groups from SAM to cytosine bases at their fifth carbon (C-5). This generates 5-methylcytosine (5-mC) that represses gene transcription (8, 11, 12) (Figure 2). 5-mC marks are most stable when they occur at CpG dinucleotides and thus are highly abundant in CpG islands (11). DNMT1 functions to maintain existing methylation patterns after DNA replication, while DNMT3a and DNMT3b induce *de novo* methylation (13). DNA methylation is dependent on the SAM/SAH ratio, partly because it relies on SAM availability. Additionally, due to the competitive inhibition of methyltransferases by SAH, DNMT function is dependent on the efficient hydrolysis of SAH by AHCY (8). Moreover, homocysteine re-methylation occurs primarily *via* the folate cycle and is necessary for the hydrolysis of SAH and regeneration of SAM. Thus, an adequate folate supply is required for efficient DNA methylation (8).

DNA demethylation, or the removal of 5-mC, occurs through base modifications catalyzed by ten-eleven translocation (TET) enzymes or activation-induced deaminase (AID) (14, 15). In TET-catalyzed base modification, TET 5-mc dioxygenase 1 (TET1) oxidizes 5-mC into 5-hydroxymethylcytosine (5-hmC) (8, 11) (Figure 2). TET1, TET2, and TET3 can subsequently oxidize 5-hmC into 5-formylcytosine and 5-carboxylcytosine (16). AID-catalyzed base modifications are less understood but are thought to involve AID (a member of the AID/APOBEC family of cytidine deaminase) acting on an unmodified cytosine in the vicinity of a 5-mC mark (14, 15). Both TET-catalyzed and AID-catalyzed base modifications are followed by nucleotide replacement *via* the base excision repair (BER) pathway (13–15).

## Alcohol induces DNA hypomethylation in the liver

By altering the activity and expression of enzymes involved in one-carbon metabolism, such as methionine synthase, methionine adenosyltransferase, and betaine-homocysteine methyltransferase, alcohol consumption decreases the methylation index in the liver (16–22). As ethanol is metabolized, acetaldehyde—one of its breakdown products—irreversibly inhibits methionine synthase (MS) (16–18). Further, alcohol downregulates the mRNA expression of MTHFR and certain MAT subunits (9, 16, 19). The reduced activity of these enzymes impairs the methionine cycle’s efficiency, leading to reduced SAM availability (20). Because alcohol also downregulates the mRNA expression of betaine-homocysteine methyltransferase (BHMT), the betaine pathway is unable to generate enough SAM to compensate (19, 21, 22).

Ethanol also reduces dietary folate absorption by altering the binding and transport kinetics of the folate transport system and reducing the expression of folate transporters in the intestines and kidneys (23, 24). This folate deficiency, in addition to the inhibition of MS by acetaldehyde, contributes to the increased plasma homocysteine levels commonly seen in association with alcohol consumption (24–26). High homocysteine levels can cause the SAH hydrolysis reaction kinetics to shift in favor of the reverse reaction, leading to a build-up of hepatic SAH and a decreased SAM/SAH ratio (20, 27, 28).

Hyperactivity of the transsulfuration pathway also contributes to this reduced hepatic methylation index. This pathway is highly active in the liver under normal conditions, but it becomes even more active during chronic alcohol consumption to attenuate oxidative stress. Because the transsulfuration pathway consumes SAM without replenishing methionine, its hyperactivity leads to reduced SAM availability (29). This, in addition to the inhibition of key folate cycle and betaine pathway enzymes and increased plasma homocysteine levels due to alcohol consumption, reduces the SAM/SAH ratio, resulting in hypomethylation of genes in the liver.

The dysregulation of one-carbon metabolism and DNA hypomethylation caused by alcohol is implicated in the development of alcohol-induced liver disease (ALD) (21, 27, 29). During chronic heavy drinking, the SAM/SAH ratio decreases (20,27–29). Because the transsulfuration pathway requires SAM, the eventual depletion of SAM levels reduces the pathway’s efficacy. As a result, the liver becomes susceptible to alcohol-induced oxidative stress, which is involved in the pathogenesis of ALD (29). Folate deficiency has also been implicated in the acceleration of ALD onset, because it contributes to both SAM-deficiency and hyperhomocysteinemia (10, 27, 29). Because homocysteine is highly toxic and enhances the vulnerability of cells to oxidative injury, hyperhomocysteinemia is associated with the development of ALD (21, 26). Chronic ethanol consumption has also been shown to cause reduced expression of TET1, and the resulting decrease in DNA hydroxymethylation can contribute to hepatocyte apoptosis, ALD progression, and the development of hepatocellular carcinoma (HCC) (30–32).

Interestingly, pharmacological restoration of effective one-carbon metabolism after chronic alcohol consumption may treat alcohol-induced liver injury. Exogenous SAM administration can compensate for depleted hepatic SAM levels and effectively attenuates oxidative stress and inflammation (29, 33). In a study of human patients with alcohol-induced cirrhosis, SAM treatment was shown to improve their likelihood of survival and delay their need for liver transplantation (29). Betaine administration is also an effective treatment for ALD. It indirectly attenuates oxidative stress and apoptosis not only by increasing hepatic SAM but also by decreasing homocysteine levels (20, 29). Because the betaine pathway metabolizes homocysteine into methionine, betaine administration may be more effective than SAM administration in treating ALD (34). Additionally, betaine administration has been shown to both attenuate and reverse triacylglycerol accumulation in the liver (otherwise known as fatty liver), even despite continued ethanol consumption (27, 29).

## Alcohol-induced changes in global DNA methylation in the brain

Studies of various brain regions have shown that alcohol-induced dysregulation of one-carbon metabolism can result in an increased methylation index in the brain (16, 19). In the rat and human cerebellum, alcohol consumption was found to be associated with decreased SAH levels and increased levels of adenosylhomocysteinase (AHCY), which facilitates the conversion of SAH into homocysteine (16, 19). These changes increase the SAM/SAH ratio and reduce the feedback inhibition of transmethylation reactions by SAH. In the human cerebellum, alcohol consumption was also found to be associated with increased expression of MTHFR, which promotes the formation of 5-meTHF for use in the conversion of homocysteine to methionine (16). Together, these effects of alcohol on one-carbon metabolism enzymes result in greater SAM availability and a higher potential for DNA methylation in the cerebellum.

Alcohol consumption also modulates the expression of enzymes directly involved in DNA methylation (16, 35). Interestingly, decreased TET1 expression was associated with alcohol consumption in humans, resulting in DNA hypermethylation (16). Further evidence has demonstrated that alcohol-induced hypermethylation is associated with upregulated DNMT1 and can be prevented by infusion of a DNMT inhibitor (35). It is possible that the magnitude of hypermethylation may depend on total alcohol consumption. A study of rhesus macaques found a positive correlation between CpG methylation and daily average alcohol intake (36); however, more studies are needed to confirm this finding. In summary, alcohol consumption is associated with the altered expression of enzymes central to one-carbon metabolism and DNA methylation, and these changes promote a global state of DNA hypermethylation in the brain (16, 19, 35, 36).

## Gene-specific DNA methylation and altered neurobiology in AUD

Studies have identified many genes that are differentially methylated in association with alcohol consumption. The resulting changes in expression of these genes underlie a variety of phenotypes related to acute and chronic alcohol consumption, dependence, and withdrawal (4). GABAergic signaling regulates motor coordination, which is known to be disrupted by alcohol consumption. One study found decreased mRNA and protein expression of GABRD, the δ subunit of the GABA_A_ receptor, in human AUD patients compared to controls (16). This was associated with hypermethylation of the *GABRD* promoter DNA. Additionally, *GABRD* mRNA expression was negatively correlated with number of total years the patients spent consuming alcohol. This decrease in GABRD *via* DNA methylation may be a component of the mechanism by which alcohol consumption impairs motor coordination (16).

Compulsive behavior during withdrawal is involved in the pathogenesis of AUD (35, 37, 38). In addition to global DNA hypermethylation and increased DNMT1 expression, Barbier et al. observed increased DNMT1 expression and downregulation of *Syt2*, a synaptic protein gene, in the medial prefrontal cortex (mPFC) of AUD modeled rats compared to controls (35). Infusion of DNMT inhibitor RG108 restored *Syt2* expression, and lentiviral inhibition of *Syt2* was found to increase aversion-resistant alcohol drinking, a compulsive behavior (35). Thus, these studies suggest that withdrawal-induced methylation of *Syt2* may be implicated in the induction of compulsive behaviors and subsequent risk of AUD (35, 37, 38).

Alcohol consumption increases the activity of the endogenous opioid system (EOS), which alters reward sensitivity and cognitive control over addiction-related behaviors (39). One study identified increased prodynorphin (*PDYN*) expression in the postmortem dorsolateral prefrontal cortex (dlPFC) of AUD subjects. Interestingly, AUD subjects were found to have increased methylation of the C variant of 3′-UTR SNP rs2235749 (a *PDYN* CpG SNP known to be associated with AUD) in the dlPFC compared to control subjects, and this methylation was positively correlated with dynorphin expression. Thus, methylation of this CpG SNP may be part of the mechanism by which alcohol increases *PDYN*, suggesting that individuals with the C variant may be more vulnerable to developing AUD (39).

**FIGURE 3:**
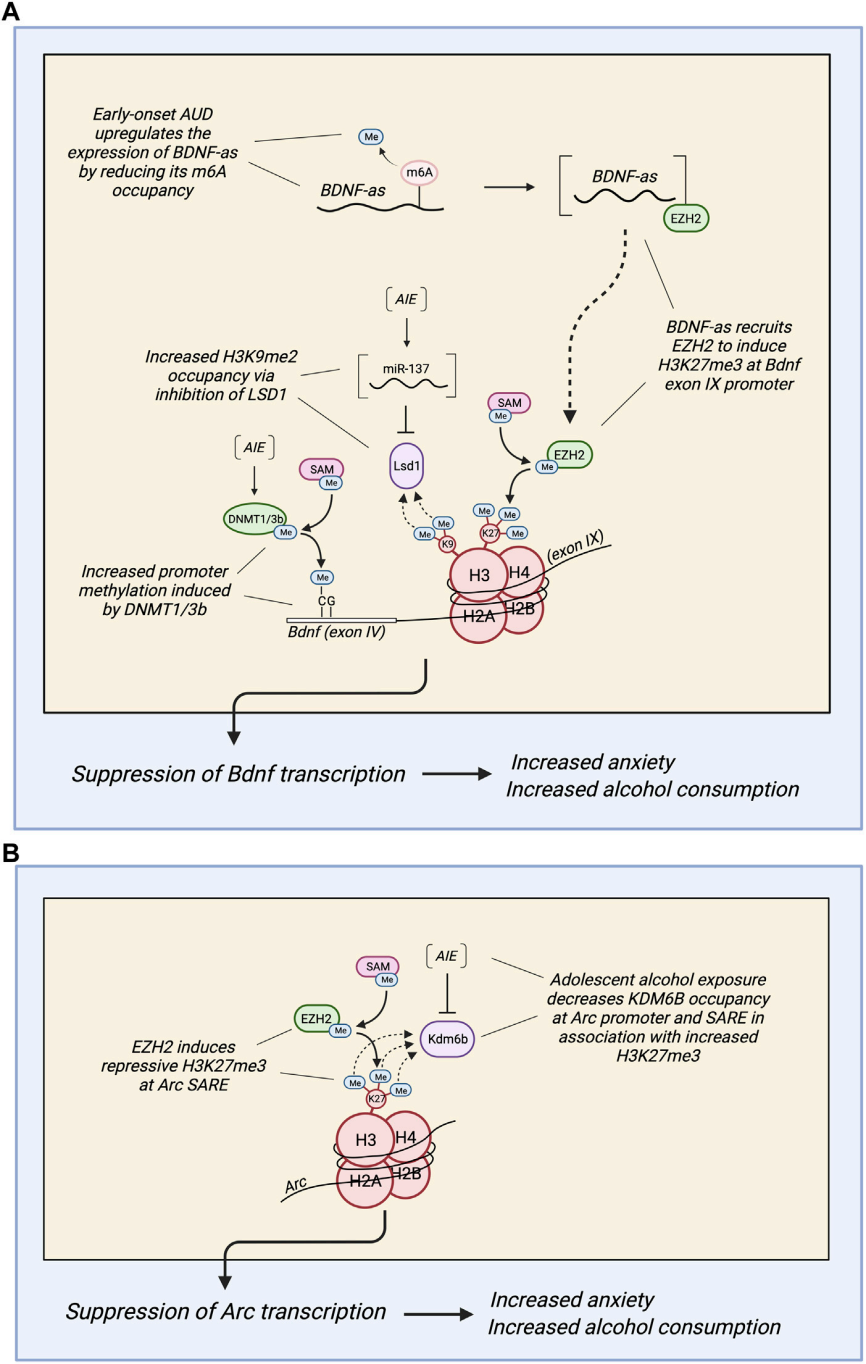
**(A)** Diagram depicting different epigenetic methylation mechanisms by which adolescent alcohol exposure regulates the expression of brain-derived neurotrophic factor (BDNF) in the adult amygdala of rats and humans. Adolescent intermittent ethanol (AIE) exposure produces hypermethylation of the *Bdnf* exon IV promoter *via* increased DNA methyltransferase activity (most likely due to increased expression of DNMT1/3b). AIE also increases H3K9me2 occupancy at the *Bdnf* exon IV promoter by upregulating miR-137, which inhibits lysine-specific histone demethylase 1 (*Lsd1*) (13,43). Further, AUD subjects with early age of onset are associated with reduced *N6*-methyladenosine (m6A) occupancy in *BDNF* antisense (*BDNF-AS*) RNA, increasing its expression. This most likely increases *BDNF-AS* recruitment of enhancer of zeste homolog 2 (EZH2) to induce repressive H3K27 trimethylation (H3K27me3) at the *BDNF* exon IX promoter and overlapping region (44). Together, these epigenetic marks induced by adolescent alcohol exposure (DNA hypermethylation, increased H3K9me2, and increased H3K27me3) suppress BDNF in the adult amygdala, leading to increased anxiety and alcohol consumption in adulthood. **(B)** Diagram depicting a mechanism by which adolescent alcohol exposure reduces the expression of *Arc* in the adult amygdala of rats and humans. AIE decreases lysine demethylase 6b (KDM6b) occupancy at the *Arc* promoter and SARE, which permits increased H3K27me3 at the Arc promoter and SARE site (45). In AUD subjects with early age of onset, there is an increase in EZH2 and H3K27me3 occupancy at the *Arc* SARE site that is associated with decreased Arc expression (44). Also, suppression of *Arc* expression in the adult amygdala increases anxiety and alcohol consumption in adulthood after adolescent alcohol exposure.

While alcohol consumption has anxiolytic effects, withdrawal from alcohol consumption results in increased anxiety. This subsequently promotes further drinking to reduce anxiety (1, 2, 3). A similar phenotype is observed in adults who first consumed alcohol during adolescence. They tend to experience higher levels of anxiety compared to alcohol-naïve adults, and this predisposes them to AUD (13). Neuropeptide Y (NPY) and brain-derived neurotrophic factor (BDNF) exert anxiolytic functions in the amygdala, and their downregulation has been observed in association with increased anxiety and alcohol consumption after adolescent alcohol exposure (13, 40–42). One study found that adolescent intermittent ethanol (AIE) exposure led to increased DNA methylation at the *Npy* promoter and *Bdnf* exon IV promoter in the adult rat amygdala in comparison to control rats, presenting a potential mechanism for the established AIE-induced downregulation of NPY and BDNF (13, 40). This methylation was associated with increased DNMT activity, higher mRNA expression of *Dnmt1* and *Dnmt3b*, and lower mRNA expression of *Gadd45g* (a protein involved in demethylation) in the adult amygdala of AIE rats compared to controls (Figure 3A). Further, the AIE adult rats exhibited heightened anxiety-like and alcohol drinking behaviors compared to control rats. In support of these results, treatment with 5-azacytidine (a DNMT inhibitor) reversed the AIE-induced increases in anxiety-like behaviors and alcohol consumption and normalized the methylation status of the *Npy* and *Bdnf* promoters. Thus, adolescent alcohol exposure may lead to psychopathology associated with reduced NPY and BDNF in adulthood *via* DNA methylation of *Npy* and *Bdnf* (13, 40–42).

Alcohol-induced epigenetic modifications of genes involved in glutamatergic and GABAergic signaling have a variety of effects, such as altered synaptic plasticity and withdrawal excitotoxicity, which increase an individual’s risk of developing AUD. GPR39 is involved in the regulation of neurotransmitter release, and JAKMIP1 contributes to neurotransmitter trafficking. Hypermethylation of the GPR39 and JAKMIP1 promoter regions, in association with decreased protein expression, was observed in the NAc of rhesus macaques after chronic self-administration of alcohol. Interestingly, these effects were correlated with average daily alcohol consumption (36). Macaques with heavy levels of consumption were found to have significantly greater DNA methylation and lower protein expression of GPR39 and JAKMIP1 compared to those with low levels of consumption (36).

Alcohol consumption is also associated with altered NMDA receptor 2B (*NR2B*) expression that may occur *via* DNA methylation (46). Demethylation at the 5′ regulatory region of the *NR2B* gene was observed in association with increased *NR2B* expression in primary cortical cultured neurons after chronic intermittent ethanol treatment. The demethylation sites were located near transcription factor binding sequences for AP-1 and CRE. Interestingly, these effects persisted during withdrawal (46). This change does not reflect the established ethanol-induced global hypermethylation in the brain, but rather may occur as a neuroadaptive response to the chronic blockade of NMDA receptors by ethanol. Alternatively, other authors have suggested that because NR2B over-expression persists even when ethanol is no longer present to block NMDA receptors, the subsequent increase in excitatory neurotransmission results in withdrawal-associated excitotoxicity (47). In support of this hypothesis, it has been shown that treatment with NMDA receptor antagonists (e.g., MK-801, CGP-39551, and dizocilpine) reduces withdrawal symptoms in different models, while treatment with NMDA receptor agonists (N-methyl-D-aspartate and kainic acid) intensifies them (47).

Alcohol-induced methylation also contributes to excitatory syndrome in withdrawal *via* homocysteine. Because chronic ethanol consumption disrupts the blood-brain barrier, the brain is exposed to elevated homocysteine levels caused by alcohol-induced hypomethylation in the rest of the body. Because homocysteine is an excitatory amino acid, it activates metabotropic glutamate receptors (mGluRs) and NMDA receptors, causing increased glutamatergic neurotransmission. Thus, the persistent elevation of blood homocysteine levels due to ethanol contributes to the brain’s risk of hyperexcitation (25, 47). In addition to its excitotoxic effects, homocysteine is also associated with brain damage and neurodegeneration. In patients with AUD, a plasma homocysteine levels were found to correlate with reductions in hippocampal volume. Plasma homocysteine levels have also been found to correlate with brain atrophy in elderly individuals. Further, homocysteine inhibits the expression and synthesis of antioxidant enzymes and radical scavengers, which results in increased cellular apoptosis in response to oxidative stress (21, 26, 47). Thus, exposure of the brain to elevated homocysteine levels leads to overstimulation of NMDA receptors, oxidative stress, DNA damage, and endoplasmic and mitochondrial dysfunction, which may be responsible for some of the brain damage and neurodegeneration associated with AUD (21, 25, 26, 47).

The mesolimbic dopamine (DA) system has been implicated in many facets of AUD, such as craving, impulse control, seeking behavior and reward salience (48). Studies of humans, primates, and rodents have found that chronic ethanol treatment leads to reduced mesolimbic DA release (49–51). These changes in DA signaling may occur *via* alcohol-induced DNA methylation of key regulatory genes (52–61). Dopamine receptor D2 (D2R) (*DRD2*) is an inhibitory receptor that is activated in response to negative outcomes to reduce DA signaling. The downregulation of D2R results in impaired impulse control and behavioral disinhibition (52). D2R is also involved in the activation of the synaptic dopamine transporter (DAT) to attenuate high synaptic DA levels by increasing reuptake. Thus, when D2R is downregulated, the resultant decrease in DAT activation leads to hyperexcitability of DA pathways in response to rewarding stimuli. One study identified hypermethylation of *DRD2* in the saliva of human AUD subjects compared to controls. *DRD2* methylation was found to correlate with clinical measures of AUD severity, as well as with reward sensitivity to alcohol over appetitive cues (53). This evidence suggests that methylation of *DRD2* may be implicated in the development of reward-related phenotypes associated with AUD.

Because of D2R’s role in the activation of DAT, the association between reduced D2R expression and alcohol consumption supports the possibility of a similar relationship between alcohol consumption and DAT expression (53). Interestingly, studies of DAT expression and methylation after chronic alcohol consumption have produced conflicting results. Several studies have found that chronic ethanol treatment causes decreased methylation of the *DAT1* promoter, resulting in the upregulation of DAT in a variety of brain regions (54–56). However, other studies have demonstrated the opposite effect, finding that chronic alcohol consumption reduces mesolimbic DAT in association with hypermethylation of the *DAT1* promoter (57–59). Some studies have also found decreased DAT expression to be associated with increased alcohol craving and voluntary alcohol consumption (60, 61).

While this evidence is still inconclusive, the possible hypermethylation of *DAT1* by chronic ethanol consumption would be consistent with the decreased mesolimbic DA release associated with chronic drinking (50–52). In this condition of reduced DA release, *DAT1* hypermethylation and decreased DAT expression could serve as a compensatory mechanism to maintain extracellular dopamine levels by decreasing synaptic reuptake (57, 62, 63). This would increase the sensitivity of the DA pathway to dopamine release, which could serve to potentiate the reward value of alcohol during withdrawal (60, 61). However, though this hypothesis is consistent with the known effects of alcohol on the DA system, more conclusive evidence is needed.

As a stressor, acute alcohol consumption induces glucocorticoid secretion, which increases the level of cortisol in the blood (64–66). As AUD develops, allostatic regulation of the hypothalamic pituitary adrenal (HPA) axis attenuates the accompanying chronically elevated cortisol levels, resulting in reduced cortisol responsivity to stress (1, 2, 66). The mechanism of this alcohol-induced allostatic regulation may involve modulation of glucocorticoid receptor (GR) expression. One study identified increased promoter methylation at exon 1_H_ of *NR3C1*, which encodes GR, in postmortem brain tissue of human AUD subjects compared to controls (64). This hypermethylation was associated with reduced mRNA and protein GR expression in the hippocampus, amygdala, striatum, and PFC (Brodmann Area 10), as well as altered expression of several other stress-responsive genes in the PFC (64). This evidence suggests that the body’s allostatic mechanism for tolerance to chronic alcohol-induced glucocorticoid secretion involves GR downregulation *via* methylation of *NR3C1*. Additionally, because *NR3C1* also serves to enhance *DRD2* expression, this evidence is consistent with the previously discussed downregulation of DRD2 in AUD (53, 67).

## Histone methylation

Methylation of DNA and histones is often locally coordinated to exert concordant effects on gene transcription (68–70). Like DNA methylation, histone methylation is dependent on SAM (5, 7). However, histone methylation does not universally increase or decrease based on the methylation index. Rather, because histone methylation marks can be repressive or activating, they are often deposited in such a way that reinforces the transcriptional effects of a particular gene’s DNA methylation status.

Methylation can occur on lysine residues in histones H3 and H4, and each lysine can be mono-, di-, or tri-methylated (71–73). Lysine methylation is regulated by methyltransferases (KMTs or “writers”) and demethylases (KDMs or “erasers”) (74). These enzymes are highly specific to individual lysine residues and particular degrees of methylation (72–74). Effector proteins (or “readers”) are responsible for recognizing a methylated lysine site and exerting the corresponding effect on gene expression (75).

The primary KMT class is SET-domain-containing enzymes (74, 75). Two of these, G9a and GLP, form a functional complex through their SET domains and can induce methylation at H3K9 and H3K27 (76). The G9a–GLP complex is particularly associated with the induction of H3K9me2, which functions to downregulate gene expression (76). Other SET family KMTs are MLL1 and MLL2, both of which induce methylation at H3K4 (56). Additionally, enhancer of zeste homolog 2 (EZH2) induces repressive trimethylation of H3K27 (H3K27me3) (42).

One important KDM is lysine-specific histone demethylase 1 (LSD1 or KDM1A/B) (40, 71, 72). LSD1 reverses H3K4me1/2 and H3K9me1/2 *via* an amine oxidase reaction using flavin as a cofactor (40, 71). Several other KDMs are Jumonji C domain-containing (JmjC) demethylases, or JHDMs (40, 72, 74). JHDM1 demethylates H3K36, JHDM2A-D and JHDM3 demethylate H3K9 and H3K36, and JARID1C (JmjC AT-rich interactive domain 1C enzyme) demethylates H3K4me2/3 (40, 77).

**FIGURE 4:**
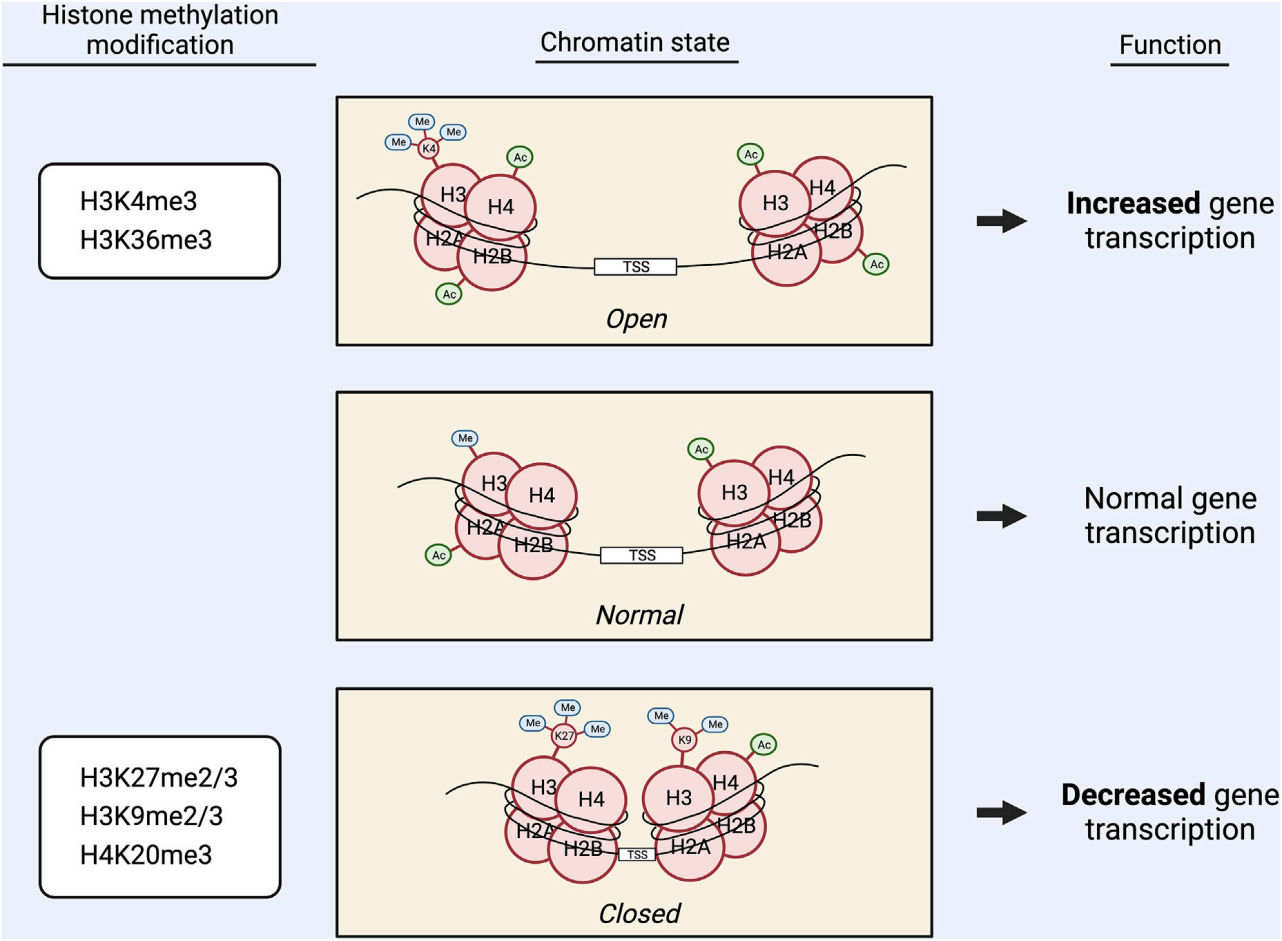
Graphic displaying the effects of methylation of different lysine (K) residues in histones H3 and H4 on chromatin state and gene transcription. Activating marks, including H3K4me3 and H3K36me3, induce a more open chromatin state, which promotes gene transcription. Repressive marks, including H3K27me2/3, H3K9me2/3, and H4K20me3, induce a more closed chromatin state, which decreases gene transcription. Alternatively, certain mono-methylation marks, such as H3K9me, H3K36me, and H3K4me, are capable of exerting both activating and repressive effects on gene transcription.

Histone lysine methylations have differential effects on gene expression (Figure 4): They can be found at both active and inactive open reading frames depending on the specific lysine residue and degree of methylation (73, 74). Activating marks typically include H3K4me3, and H3K36me3, whereas H3K27me2/3, H3K9me2/3, and H4K20me3 are typically repressive (72, 75, 76). However, certain mono-methylation marks—such as H3K9me, H3K36me, and H3K4me—can cause activation or repression of gene expression (77, 78) (Figure 4).

## Alcohol alters histone methylation in the liver

Though chronic alcohol consumption decreases the methylation index in the liver, this does not result in a global decrease in histone methylation as with DNA methylation. Instead, chronic alcohol consumption results in histone methylation changes that reinforce the transcription-promoting effects of global DNA hypomethylation: increased activating marks and decreased repressive marks (79, 80). This was demonstrated in hepatocytes treated with ethanol, which showed reduced repressive mark, H3K9me2, and increased active mark, H3K4me2, in association with a two-fold increase in about 35% of the genes expressed in the liver (79, 80). This evidence suggests that alcohol’s effect on histone methylation in the liver serves to contribute to a global state of increased gene transcription, in concordance with alcohol-induced DNA hypomethylation (79, 80).

Alcohol-induced changes in histone methylation, as with DNA methylation, contribute to the pathophysiology of ALD. Alcohol has been found to increase the hepatic activity of MLL1, a SET-domain-containing KMT that induces H3K4me2/3 in hepatic stellate cells. This results in accelerated trans-differentiation of these cells, leading to increased net deposition of fibril-forming extracellular matrix. This process eventually leads to fibrosis, a common feature of ALD (81).

Thus, alcohol-induced changes in histone methylation in the liver mirror those of DNA methylation. Though histone methylation is not globally reduced in the liver, it is still altered in such a way that reinforces the transcription-promoting effects of DNA hypomethylation: Repressive marks are decreased, and activating marks are increased. Further, alcohol’s effects on histone methylation contribute to the development of ALD, in combination with its effects on DNA methylation (79–81).

## Alcohol induces specific changes in histone methylation in the brain

Alcohol consumption induces a variety of changes in histone methylation that contribute to phenotypes associated with the pathophysiology of AUD. As previously discussed, the EOS of AUD individuals is characterized by increased PDYN because of methylation at particular SNPs in the *PDYN* promoter, which promotes anxiogenesis (39). Another study found that acute alcohol caused increased DYN and NOC in association with increased H3K9Ac and decreased H3K27me3 at the *Pdyn* and *Pnoc* promoters in the amygdala of treated rats compared to controls. This may reflect the role of acute alcohol as both an anxiolytic and an acute stressor (82–84).

As previously discussed, DNA methylation is implicated in the induction of anxiety in adult rats that were exposed to alcohol during adolescence (Figure 3A) (13, 40). Studies have demonstrated similar effects of AIE on histone methylation (43). Decreased *Arc* eRNA expression was observed in the amygdala of adult AIE rats compared to controls. This was associated with increased anxiety-like behaviors as well as decreased KDM6B occupancy and increased H3K27me3 occupancy at the *Arc* synaptic activity response element (SARE) site and promoter. Interestingly, knockdown of KDM6B *via* siRNA infusion induced the same effects in alcohol-naïve adults: increased anxiety-like behaviors, decreased *Arc* eRNA and mRNA expression, decreased KDM6B occupancy, and increased H3K27me3 occupancy at *Arc* regulatory sites (43). This evidence suggests that AIE leads to increased H3K27me3 at the *Arc* promoter by downregulating the activity of KDM6B, causing decreased *Arc* expression and increased anxiety-like behaviors in adulthood (Figure 3B). A subsequent study confirmed this epigenetic remodeling using targeted epigenomic editing (85). Infusion of dCas9-P300 into the CeA increased H3K27Ac at *Arc* SARE, increased *Arc* eRNA and mRNA expression, and ameliorated the AIE-induced anxiety and excessive alcohol intake in adult rats. Further, infusion of dCas9-KRAB into control rats led to increased repressive H3K27me3 at *Arc* SARE, decreased *Arc* eRNA and mRNA expression, and the development of anxiety and alcohol drinking behaviors (85). Thus, these findings causally link epigenetic modifications of *Arc* at H3K27 with the induction of anxiety and alcohol consumption phenotypes in adulthood as a result of AIE (85).

In addition to DNA hypermethylation, histone methylation changes also contribute to the AIE-induced downregulation of *Bdnf*. A study found decreased *Bdnf* expression in the amygdala of AIE adult rats compared to controls in association with increased H3K9me2 occupancy at the *Bdnf* exon IV promoter. This was also associated with decreased *Lsd1* and *Lsd1+8a* mRNA expression, decreased LSD1 protein expression, and decreased LSD1 binding to the *Bdnf* exon IV promoter, as well as increased anxiety-like behaviors and voluntary ethanol consumption (40). A subsequent study demonstrated increased expression of miR-137, a microRNA that targets *Lsd1*, in the amygdala of AIE adult rats compared to controls (40, 41). Exposure of AIE adult rats to acute ethanol normalized the AIE-induced phenotypes to those of control rats: the heightened anxiety-like behaviors were attenuated, the decreased *Lsd1+8a* mRNA expression was normalized, and the increased H3K9me2 occupancy at the *Bdnf* exon IV promoter was normalized (40). Interestingly, infusion of a miR-137-specific antagomir into the CeA also reversed these AIE-induced phenotypes: anxiety-like behaviors were attenuated, voluntary ethanol consumption was normalized, the decreased *Lsd1* and *Lsd1+8a* mRNA expression was rescued, the decreased LSD1 binding to the *Bdnf IV* promoter was restored, the increased H3K9me2 occupancy at the *Bdnf IV* promoter was normalized, and the decreased expression of *Bdnf* was rescued. Notably, co-infusion of *Lsd1* siRNA prevented the normalization of these phenotypes, indicating that LSD1 is an essential component of this mechanism (41). In summary, these results suggest that AIE increases miR-137 in the adult amygdala, which allows increased H3K9me2 occupancy at the *Bdnf* exon IV promoter *via* the inhibition of *Lsd1*. This modulation, in addition to the AIE-induced DNA hypermethylation of the *Bdnf* exon IV promoter, results in reduced BDNF, which contributes to the development of anxiety-like behaviors and increased voluntary alcohol consumption in adulthood (13, 40, 41) (Figure 3A). In addition to its role in AIE-induced adult anxiety, NPY is also involved in the anxiolytic effects of acute ethanol (13, 86). Decreased H3K9me2 and G9a occupancy at the regulatory region of *Npy* has been observed in rats treated with acute ethanol in association with increased NPY expression and decreased anxiety-like behaviors (86). Thus, altered histone methylation of NPY appears to be involved in anxiolysis due to acute ethanol.

As previously discussed, increased NR2B expression is implicated in multiple phenotypes of AUD. In addition to DNA hypomethylation of the *NR2B* promoter, histone methylation changes also appear to contribute to the alcohol-induced upregulation of NR2B. One study demonstrated that chronic intermittent ethanol exposure causes increased H3K9ac and decreased H3K9me2/3 occupancy at the *NR2B* promoter in a cortical neuronal cell culture, resulting in increased NR2B expression. This was observed in association with decreased expression of multiple KMTs, including G9a (87).

Alcohol may also affect GABAergic signaling by modulating the expression of GABA-Aɑ5, a subunit of GABAA. Studies have shown that GABAA receptor activity is associated with a variety of alcohol tolerance, dependence, and withdrawal phenotypes, and GABA-Aɑ5 has been implicated in alcohol-induced memory loss and cognitive impairment (16, 88–90). Increased GABA-Aɑ5 expression has been observed in association with increased H3K4me3 and H3K9ac occupancy in the PFC of rats treated with chronic ethanol compared to controls (88). Interestingly, increased GABA-Aɑ5 expression and H3K4me3 occupancy has also been observed in rats with a genetic background of AUD, and this was associated with increased vulnerability to alcohol addiction. Because of this association, alcohol-induced upregulation of GABA-Aɑ5 *via* histone methylation may increase the risk that an individual and their offspring will develop AUD (88–90).

One study identified increased expression of four genes related to ethanol tolerance (HSP104, PRO1, TPS1, and SOD1) in ethanol-tolerant strains of *S. cerevisiae* in association with enhanced H3K4me3 occupancy at the promoter of each (91). After passage through a stress-free medium treatment, the expression of these genes and H3K4me3 occupancy was significantly decreased. This evidence suggests that H3K4me3 is involved in mediating the ethanol tolerance of these strains (91). Another study also found evidence supporting the role of histone methylation in ethanol tolerance (92). Individual knockout of four JHDMs (NO66, KDM3, KDM5B, and HSPBAP1) in *drosophila* resulted in differential changes in ethanol tolerance and sensitivity to ethanol-induced sedation (92, 93). Thus, these studies suggest that histone demethylases may be required for normal ethanol tolerance and ethanol-induced sedation (91–93).

## RNA methylation

While the involvement of epigenetic mechanisms in the pathophysiology of AUD has been extensively characterized (3,4), far less is known about the role of epitranscriptomics (RNA epigenetics). Broadly, epitranscriptomics describes molecular modifications of RNA, including messenger RNA, transfer RNA, ribosomal RNA, and non-coding RNAs, that exert functional changes (94). There are many known RNA modification types, but the most widely studied involve the methylation of guanine, cytosine, and adenine. As compared to those of guanine and cytosine, adenine modifications have been the most extensively investigated. Adenine can be methylated in diverse ways, but the most prevent is the m6A modification, which accounts for approximately 50% of all methylated ribonucleotides (94). M6A modifications are reversible and have been shown to control splicing, stability, and translation of the transcriptome (94, 95). Strikingly, the mechanism of m6A methylation also involves the usage of SAM, which is part of one-carbon metabolism. This connection suggests m6A methylation also plays an important role in the fine tuning of gene expression in AUD, and understanding how cells manage their one-carbon metabolism and output of SAM for the all methylation mechanisms discussed herein are perhaps indispensable to developing treatments for AUD. Furthermore, the m6A methylation could be under the control of epigenetic mechanisms, and such a feature would provide a mechanistic bridge between gene regulation and gene expression (95). Thus, m6A modified RNAs, especially mRNAs, present an enticing avenue for exploration into the pathophysiology of AUD.

**FIGURE 5:**
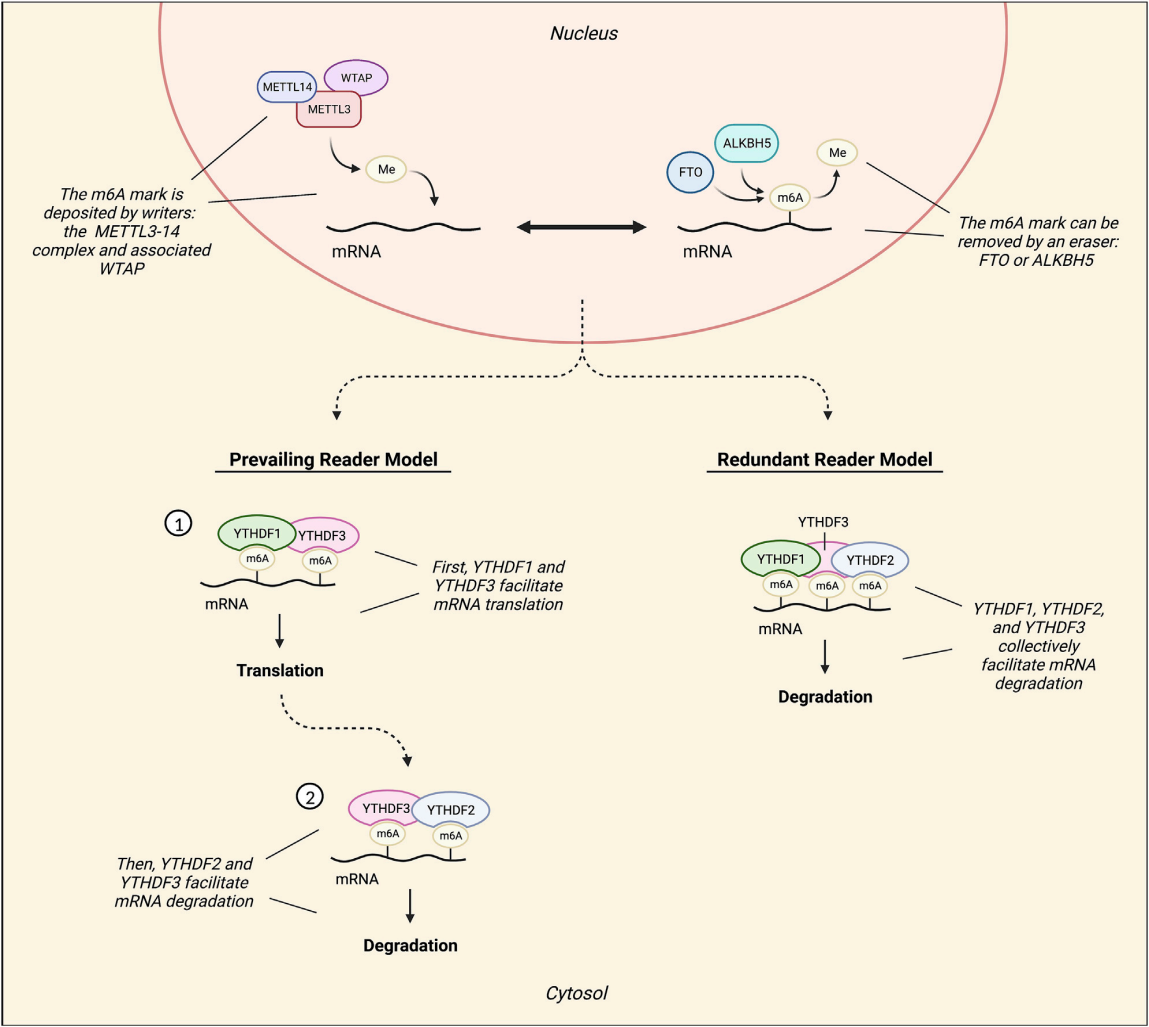
Diagram illustrating the mechanisms involved in RNA m6A methylation. The METTL3-14 methyltransferase (writer) complex, which can be assisted by ancillary writers such as Wilm’s tumor 1-associating protein (WTAP), confers m6A methylation marks. Conversely, demethylases ALKBH5 and FTO remove m6A marks. Readers are responsible for processing and defining the fate of m6A RNAs. The most characterized readers are YTHDF1, YTHDF2, and YTHDF3, and there are two contrasting models of their functionality: the prevailing model and the redundant model. According to the prevailing model, an m6A mark is first read by YTHDF3 with the assistance of YTHDF1. YTHDF1 then recruits translation factors to promote the translation of the marked mRNA. Following this, the transcript is inevitably read by YTHDF2 under facilitation by YTHDF3 to promote its decay. Conversely, the redundant reader model suggests that instead of exerting independent, disparate functions on marked transcripts, these readers interact with marked mRNA transcripts collectively to promote its degradation.

M6A methylation is conferred to RNAs co-transcriptionally by the METTL3–METTL14 methyltransferase (writer) complex, and its associated ancillary proteins. METTL3 (methyltransferase-like 3) is the catalytically active subunit, while METTL14 (methyltransferase-like 14) functions as a holding scaffold that facilitates RNA binding to the complex (96–98). Of the ancillary writers, Wilm’s tumor 1-associating protein (WTAP) is the most notable, and it is known to recruit the METTL3-14 complex to nuclear speckles (97, 98) (Figure 5). Although some m6A sites are WTAP-dependent and others are WTAP-independent, knockdown of WTAP is known to drastically reduce m6A mRNA levels (94, 98).

Two m6A demethylases (erasers) have been characterized thus far, ALKBH5 and FTO (Figure 5). ALKBH5 directly removes the methyl group from an m6A mark without oxidizing (97, 98). FTO, on the other hand, reverses both m6A and m6Am *via* oxidation, but has a higher catalytic activity for m6Am (96, 97). FTO is also known as the “obesity risk gene” because its dysregulation, particularly *via* common SNP variants, has been associated with obesity (99).

M6A readers process and define the fate of m6A RNAs. The most characterized group of m6A readers is the YT521-B homology domain family (YTHDF), which is comprised of YTHDF1, YTHDF2, and YTHDF3 (96–98). According to the prevailing m6A reader model, YTHDF1 functions to promote mRNA translation by recruiting various translation factors, such as eIF3, to marked mRNAs (95, 98). In contrast, YTHDF2 facilitates the localization of marked mRNA transcripts to processing bodies for degradation (94, 97, 98) (Figure 5). However, certain stress conditions can trigger the localization of marked transcripts to stress granules instead, where they are temporarily protected from degradation (94). Additionally, YTHDF2 may also function to preserve 5′UTR m6A marks on stress-induced transcripts by preventing FTO-mediated demethylation in the nucleus (98). Lastly, YTHDF3 does not exert independent effects, but rather appears to assist in the functions of both YTHDF1 and YTHDF2 through temporal patterns of interaction, first facilitating mRNA translation with YTHDF1 and then committing mRNAs for degradation with YTHDF2 (100) (Figure 5).

Conversely, an alternative model of m6A reader function has been suggested: the redundant reader model (101). This model postulates that the readers do not confer biologically significant and disparate effects independently, but rather, function cooperatively to promote m6A RNA decay (94, 101, 102) (Figure 5). In mouse embryonic stem cells, only the simultaneous knockout of all YTHDFs decreased the degradation rate of m6A mRNAs, while knockout of individual YTHDFs did not result in significantly decreased degradation rates (102). Moreover, additional X-ray crystallography and molecular dynamics data have demonstrated that the YTHDFs all interact with m6A mRNAs in the same way (103). In light of the discrepancies between this model and the prevailing m6A reader model, it appears particularly important to study all three YTHDFs collectively whenever possible.

## Implication of m6A in liver and kidney pathology

While studies are lacking on the role of m6A methylation in ALD specifically, many studies have found that m6A regulation is involved in adipogenesis, a hallmark symptom of ALD. Decreased m6A *via* oxidation by FTO has been shown to cause increased adipogenesis (104, 105). Increased *FTO* mRNA and protein levels have been observed in the liver in patients with non-alcoholic fatty liver disease (104). Further, Hu et al. found that FTO is activated by glucocorticoid receptors to erase m6A marks on the mRNA of lipogenic genes (106). Conversely, because it deposits m6A, METTL3 activity has been found to negatively correlate with adipogenesis. YTHDF2 activity also negatively correlates with adipogenesis, as it can induce the degradation of m6A-marked lipogenic transcripts (104).

In addition to liver disease, kidney inflammation is a common consequence of chronic alcohol consumption. Yu et al. identified a potential mechanism for this inflammation *via* m6A methylation. In a mouse model of alcohol-induced kidney injury, alcohol was found to cause increased DNA methylation of FTO in the kidneys, subsequently reducing its expression. This was confirmed *via* inhibition of DNMTs, which attenuated alcohol-induced kidney injury and reversed the downregulation of FTO. Alcohol was also found to cause increased YTHDF2 mRNA and protein levels in the kidneys. Based on this evidence, Yu et al. proposed that alcohol-induced downregulation of FTO increases m6A methylation of peroxisome proliferator-activated receptor alpha (PPAR-ɑ). These marks are then read by YTHDF2, destabilizing and promoting degradation of PPAR-ɑ mRNA, leading to inflammation (107). This data further suggests that knocking down METTL3 may also confer a protective effect against kidney inflammation induced by chronic alcohol consumption. However, differential regulation of the m6A proteins in various tissues is incredibly complex, so these hypothesized mechanisms require further investigation.

## Relationship between m6A and alcohol in the brain

M6A methylation appears to be another mechanism by which alcohol consumption alters BDNF expression in the amygdala. Increased levels of *BDNF* antisense (*BDNF-AS*), a long non-coding RNA (lnc-RNA) that regulates BDNF expression, have been found in the postmortem amygdala of individuals with AUD who began drinking during adolescence compared to those with late-onset AUD and control subjects (42). Interestingly, there was a significant reduction in *BDNF-AS* RNA methylation at m6A sites in adolescent-onset AUD but not in late-onset AUD. In light of the ability of m6A methylation to promote degradation and alter the recruitment of RNA-binding proteins, the authors suggest that the decrease in m6A methylation of *BDNF-AS* may play a causal role in its upregulation in adolescent-onset AUD (42). *BDNF-AS* most likely recruits EZH2 to induce H3K27me3 at the *BDNF* exon IX promoter and overlapping region, which inhibits *BDNF* expression. Because BDNF activates *ARC* transcription, *ARC* expression may also be indirectly downregulated by *BDNF-AS*. The decreased expression of both genes as a result of *BDNF-AS* upregulation is associated with increased anxiety, alcohol consumption, and risk of AUD (40, 42). This epigenetic regulation of *BDNF* and *ARC* expression in early-onset AUD is consistent with other AIE-induced modifications found in previously discussed studies in the rat model: Increased H3K27me3 occupancy at the *Arc* SARE and promoter sites, DNA hypermethylation of the *Bdnf* exon IV promoter, and increased H3K9 occupancy at the *Bdnf* exon IV promoter (13, 43, 41). These epigenetic marks all contribute to the downregulation of BDNF and *Arc*, which has been shown to result in increased anxiety and alcohol consumption (13, 42, 41) (Figure 3B).

Studies assessing the effects of the rs9939609a *FTO* variant on an individual’s risk of AUD have produced mixed results. Some have found rs9939609a to be a risk factor for alcohol dependence (AD). An analysis of two large-scale Caucasian sample groups yielded significant associations between AD and several FTO SNPs, including the rs9939609a variant (108). Similarly, an association between AD and rs8050136a, which is in complete linkage disequilibrium with rs9939609a, was found in a mixed-ethnicities sample (109). Contrarily, other studies have found rs9939609a to be a protective factor against AD. In a Caucasian sample, rs9939609a was associated with reduced alcohol consumption (110). Likewise, rs9939609a was found to be associated with less frequent drinking, increased alcohol consumed per drinking instance, and reduced overall alcohol consumption in another analysis of a Caucasian sample (111). However, the authors of this study did not control for other variables, such as BMI or age, when comparing AD subjects to controls (109). Another study found no association, whether positive or negative, between rs9939609a and alcohol intake (112).

Interestingly, alcohol consumption and AD risk may moderate the effects of FTO obesity-risk SNPs on BMI. Genetic obesity risk based on FTO SNPs has been shown to have a larger effect on BMI in infrequent drinkers: about twice as large in those who never drank alcohol compared to every-day drinkers. This effect decreased with increased alcohol consumption frequency, and there was an overall inverse correlation between alcohol intake frequency and BMI (113). Alternatively, another study found that males with a high risk of AD had overall significantly higher BMI during adolescence compared to low-risk males. Further, high-risk males who were homozygous for the obesity-associated allele of an FTO SNP had higher BMI during late adolescence compared to those who carried the minor allele (114). Although studies have produced mixed results regarding the effects of obesity-risk FTO alleles on risk of AUD, alcohol consumption and AD risk do appear to moderate the effects of obesity-risk FTO alleles on BMI. More studies are needed to discern whether the relationship between alcohol and FTO SNPs is causal, as well as whether there are bidirectional effects.

## Avenues for research on alcohol and m6A

Studies regarding the relationship between alcohol and m6A methylation are limited. However, existing literature has identified m6A methylation involvement in particular gene networks that are central to AUD pathophysiology. These networks pose avenues for research into the potential relationships between alcohol consumption and m6A methylation.

Several studies have demonstrated the involvement of FTO-mediated m6A methylation in the regulation of reward pathways, including the dopamine system (115–120). Overexpression of FTO in rats was found to cause increased expression of glutamate receptor subunit 1 (*Nmdar1*) *via* decreased m6A methylation. The resulting oxidative stress and Ca^2+^ influx led to apoptosis of dopaminergic neurons (116). In another study, inactivation of FTO in mice resulted in impaired D2/3R autoinhibition of midbrain dopaminergic neurons in association with increased m6A levels in the midbrain and striatum (115). Additionally, the rs9939609a variant of FTO has been associated with impaired D2/3R signaling and increased reward sensitivity (117, 119, 120). In light of the previously discussed dopaminergic effects of alcohol, particularly the downregulation of D2R signaling and increased reward sensitivity seen in individuals with AUD, further research should explore the effects of alcohol on FTO-mediated m6A methylation in the dopaminergic system (53).

Endogenous glucocorticoids involved in the acute stress response have been shown to modulate the expression of FTO, ALKBH5, and METTL3 *via* glucocorticoid response elements in their 5′ upstream regions, causing altered m6A levels across multiple brain regions in both humans and rodents. Interestingly, knockout of METTL3 and FTO has been shown to result in abnormal stress-coping behaviors, suggesting that the modulation of m6A by glucocorticoids plays a functional role in the stress response (121). As a stressor, acute alcohol consumption stimulates glucocorticoid secretion, causing increased blood cortisol levels (64, 65, 66). Additionally, high-risk social drinkers tend to have enhanced cortisol responsivity to stress, while AUD individuals have reduced cortisol responsivity to stress because of allostatic regulation (64, 66, 120). Considering the effect of glucocorticoids on m6A levels, it would be interesting to investigate how m6A levels are differentially affected by acute and chronic alcohol consumption, risk factors for AUD, and allostatic regulation. Further, elucidating more of the downstream effects of stress-induced modulation of m6A in the PFC and amygdala, in addition to the abnormal stress-coping behaviors observed in response to METTL3- and FTO-knockout, could provide valuable insights into their potential role in alcohol-related pathophysiology.

## Conclusion and future directions

This review has presented evidence of global and gene-specific epigenetic methylation modifications in the liver and brain produced by alcohol consumption. Though DNA, histone, and RNA methylation all rely on one-carbon metabolism for the generation of methyl groups, they are differentially affected by alcohol.

DNA methylation is directly influenced by the methylation index, which relies on the efficiency of one-carbon metabolism. In the liver, alcohol-induced dysregulation of one-carbon metabolism produces a decreased methylation index. The resulting DNA hypomethylation leads to changes in gene expression that underlie elements of the pathophysiology of ALD (9, 19, 22). Conversely, chronic alcohol consumption may cause an increased methylation index in the brain, leading to global DNA hypermethylation. The subsequent changes in gene expression contribute to several components of the brain pathophysiology of AUD that promote continued alcohol consumption, including withdrawal-induced anxiety, reduced behavioral inhibition, and increased reward sensitivity (16, 19, 35).

In contrast, histone methylation does not globally increase or decrease based on the methylation index. Rather, alcohol-induced histone methylation modifications often reinforce the transcriptional effects of alcohol-induced DNA methylation. In the liver, this occurs globally. Chronic alcohol consumption leads to an increased abundance of activating histone methylation marks (79, 80). In the brain, most effects of alcohol on histone methylation are gene-specific, resulting in changes in gene expression that are consistent with alcohol’s effects on DNA methylation and contribute to the pathophysiology of AUD (82–92).

Compared to DNA and histone methylation, far less is known about RNA m6A methylation and its relationship with alcohol consumption. Studies should explore whether alcohol modulates the expression and activity of RNA modifiers and the prevalence of m6A methylation in any global or region-specific ways. Elucidating these general effects will better inform subsequent research regarding the effects of alcohol on gene-specific m6A methylation. Several interesting avenues for such studies have already emerged from existing literature (95). Other RNA methylation marks (such as m5C, m7G, and m1A) should also be studied to develop a more complete picture of the links between alcohol consumption and RNA methylation. Insight into these relationships and their mechanisms will develop a new dimension of the field’s understanding of AUD pathophysiology, which will hopefully inform novel approaches to the treatment and prevention of AUD.

## Glossary

5-mC: 5-methylcytosine
AD: alcohol dependence
AHCY: SAH hydrolase
AIE: adolescent intermittent ethanol
ALD: alcohol-induced liver disease
ALKBH5: AlkB homolog 5
ARC: activity-regulated cytoskeleton associated protein
AUD: alcohol use disorder
BDNF: brain-derived neurotrophic factor
CRE: cAMP response element
DA: dopamine
dlPFC: dorsolateral prefrontal cortex
DNMT: DNA methyltransferase
DYN: dynorphin
EOS: endogenous opioid system
eRNA: enhancer RNA
EZH2: enhancer of zeste homolog 2
FTO: fat mass and obesity-associated protein
GABA: gamma-aminobutyric acid
GR: glucocorticoid receptor
KDM: lysine demethylase
KMT: lysine methyltransferase
M6A: N6-methyladenosine
METTL14: methyltransferase-like 14
METTL3: methyltransferase-like 3
mPFC: medial prefrontal cortex
MS: methionine synthase
NAc: nucleus accumbens
NMDA: N-methyl-D-aspartate
NOC: nociceptin
NPY: neuropeptide Y
SAH: S-adenosylhomocysteine
SAM: S-adenosylmethionine
SARE: synaptic activity response element
SNP: single nucleotide polymorphism
WTAP: Wilm’s tumor 1-associating protein
YTHDF: YT521-B homology domain family
